# Clustering of predicted loss-of-function variants in genes linked with monogenic disease can explain incomplete penetrance

**DOI:** 10.1186/s13073-024-01333-4

**Published:** 2024-04-26

**Authors:** Robin N. Beaumont, Gareth Hawkes, Adam C. Gunning, Caroline F. Wright

**Affiliations:** 1 Department of Clinical and Biomedical Sciences, Faculty of Health and Life Sciences, University of Exeter, Exeter, EX1 2LU UK; 2 Exeter Genomics Laboratory, Royal Devon University Healthcare NHS Foundation Trust, Exeter, EX2 5DW UK

**Keywords:** Constraint, Penetrance, Variant interpretation, Genomic medicine, Developmental disorders

## Abstract

**Background:**

Genetic variants that severely alter protein products (e.g. nonsense, frameshift) are often associated with disease. For some genes, these predicted loss-of-function variants (pLoFs) are observed throughout the gene, whilst in others, they occur only at specific locations. We hypothesised that, for genes linked with monogenic diseases that display incomplete penetrance, pLoF variants present in apparently unaffected individuals may be limited to regions where pLoFs are tolerated. To test this, we investigated whether pLoF location could explain instances of incomplete penetrance of variants expected to be pathogenic for Mendelian conditions.

**Methods:**

We used exome sequence data in 454,773 individuals in the UK Biobank (UKB) to investigate the locations of pLoFs in a population cohort. We counted numbers of unique pLoF, missense, and synonymous variants in UKB in each quintile of the coding sequence (CDS) of all protein-coding genes and clustered the variants using Gaussian mixture models. We limited the analyses to genes with ≥ 5 variants of each type (16,473 genes). We compared the locations of pLoFs in UKB with all theoretically possible pLoFs in a transcript, and pathogenic pLoFs from ClinVar, and performed simulations to estimate the false-positive rate of non-uniformly distributed variants.

**Results:**

For most genes, all variant classes fell into clusters representing broadly uniform variant distributions, but genes in which haploinsufficiency causes developmental disorders were less likely to have uniform pLoF distribution than other genes (*P* < 2.2 × 10^−6^). We identified a number of genes, including *ARID1B* and *GATA6*, where pLoF variants in the first quarter of the CDS were rescued by the presence of an alternative translation start site and should not be reported as pathogenic. For other genes, such as *ODC1*, pLoFs were located approximately uniformly across the gene, but pathogenic pLoFs were clustered only at the end, consistent with a gain-of-function disease mechanism.

**Conclusions:**

Our results suggest the potential benefits of localised constraint metrics and that the location of pLoF variants should be considered when interpreting variants.

**Supplementary Information:**

The online version contains supplementary material available at 10.1186/s13073-024-01333-4.

## Background

Contrary to expectation, many individuals in the population harbour predicted loss of function (pLoF) variants in genes where haploinsufficiency is known to cause highly penetrant monogenic conditions [1–3]. For example, pLoF variants in genes that cause severe developmental disorders (DD) in childhood would not be expected to be present at appreciable levels in the general adult population. Nonetheless, we and others have previously shown that thousands of individuals in the UK Biobank (UKB) carry pLoF variants in DD genes and have phenotypes consistent with incomplete penetrance or reduced expressivity, though very few individuals have DD diagnoses [4–7]. There are several possible explanations for this observation. One possibility is that genetic or environmental modifiers alter the impact of individual variants [8], such that the penetrance of pathogenic variants identified in affected families or disease cohorts may be over-estimated. An alternative explanation is that some pLoF variants in these genes do not cause loss of function, either because they are technical false positives [9] or mosaic variants or because they can be rescued through a variety of mechanisms, including alternative transcription [10], exon skipping [11], escape from nonsense-mediated decay (NMD) [12], and translation re-initiation [13]. It is important to distinguish between benign pLoF variants that produce near-normal levels of functional protein and pathogenic variants that result in substantially reduced protein products, both for estimating penetrance and interpreting diagnostic results. Indeed, the location of variants within the gene has been shown to be important for determining pathogenicity, e.g. variants escaping NMD causing pathogenic gain of function [14].

Constraint metrics derived from population variation have been extremely useful for identifying genes that are intolerant to pLoF variation [15, 16] and regions of genes that are intolerant to missense variation [17, 18]. However, the location of pLoF variants in genes has not been systematically investigated at a large scale due to a lack of sequence data on large numbers of individuals. We used cluster analysis of exome sequence (ES) data from UKB to identify genes showing distinct patterns in the location of pLoF variants. We then investigated genes showing these distinct profiles of pLoF location to determine whether they could explain the presence of such putatively pathogenic variants in a population cohort.

## Methods

### Classification of variants in the UK Biobank

Variants from exome sequencing (ES) data within the UK Biobank were called centrally by the UKB team using graphTyper [19]. We used the Ensembl VEP v104 [20] with the LOFTEE plugin [15] to annotate the variants with their predicted functional consequences. We excluded variants which were flagged for removal by UKB due to low depth based on 90% of calls having depth < 10. Within each protein-coding transcript, we grouped variants into synonymous (VEP classification “synonymous_variant”), missense (“missense_variant”), and pLoF (variants classified by LOFTEE as “LoF”). No frequency threshold was used, and variants were all counted once regardless of minor allele frequency. We further separated pLoF variants into single nucleotide variants (SNVs) and frameshift insertions or deletions (indels). For our main analyses, we considered all pLoF variants together; pLoF SNVs were used as a sensitivity analysis to ensure patterns were not driven by indels spanning quintiles of the gene. Using start and end locations for exons within each transcript from Ensembl (v105 downloaded on 01-04-2022), we calculated the relative position of each variant within the coding sequence of each transcript, considering only exonic variants (i.e. excluding splice donor and splice acceptor variants in the introns). We then divided each transcript into quintiles, ignoring exon boundaries, and for each class of variants (synonymous, missense, pLoF), we calculated the number of variants within each quintile as a proportion of the number of variants of that class based on the base pair position of the variants.

### Cluster analysis

We applied principal component analysis (PCA) to the proportion of variants in each quintile of the gene transcript, separated by variant class, using the SKLearn “decomposition.PCA” function. PCA loadings were calculated based on locations of synonymous, missense, and all pLoF variants. We then projected the PCA onto SNV pLoFs, possible pLoFs, simulations, and ClinVar variants.

We clustered the PCA profile of variants of each class within MANE Select transcripts using Gaussian mixtures, allowing seven clusters, trained using the profile of synonymous, missense, and all pLoFs. For each class of variant within each transcript, we obtained the most likely cluster, as well as the probability for its inclusion in each cluster. We projected the SNV pLoFs, possible pLoFs, simulations, MANE Plus Clinical transcripts, and ClinVar variants into the clusters to obtain their most likely cluster and probabilities. Seven clusters were chosen to allow for multiple clusters with different non-uniformly distributed variants. We performed sensitivity analyses varying the number of clusters to ensure that our results were robust to the number of clusters chosen.

### Sensitivity analyses

#### Possible locations of pLoF variants

We examined the coding sequence of each transcript and calculated the locations of all possible pLoF SNVs. We clustered these in the same way as observed pLoF variants (see below) to verify that any patterns we identified were not driven by the underlying coding sequence and the possible locations of pLoF SNVs. The clustering of these variants was compared to that of observed pLoF SNVs.

#### Simulations

To estimate the rate at which genes with a given number of variants of a given class clustered into each cluster, we used simulations to create synthetic sets of genes with varying numbers of variants. We took the relative positions of all variants in the UKB ES data and randomly selected a number of these based on the number of pLoF variants within each gene in UKB. We repeated this 10,000 times. Simulated genes were then clustered to estimate the number of genes falling into each cluster by chance.

#### ClinVar variants

We downloaded clinically annotated variants from ClinVar (02/10/2022) and calculated the proportion of pathogenic/likely pathogenic pLoF variants within each quintile of each transcript and clustered them to compare to the UK Biobank pLoF clusters.

#### Disease gene lists

We examined the clusters which pLoFs fell into in genes linked with monogenic diseases from the Gene2Phenotype [21] database to investigate whether these could elucidate the variable penetrance of these genes. Gene lists were downloaded from https://www.ebi.ac.uk/gene2phenotype/ (accessed 04-06-2021) and split into those causing severe developmental disorders (DD) and those causing later onset diseases (cancer, cardiac, eye, and skin). These were further subdivided into monoallelic (autosomal dominant) and biallelic (autosomal recessive) genes with “absent gene product” mechanisms; G2P genes with other inheritance classes or disease mechanisms were excluded.

## Results

### pLoF variants are more likely to be non-uniformly distributed than missense or synonymous variants

**Fig. 1:**
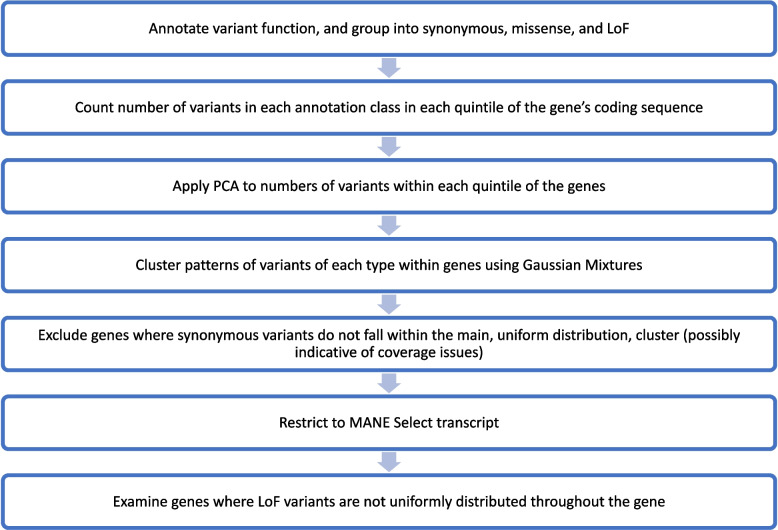
Flow diagram showing the experimental design

**Fig. 2:**
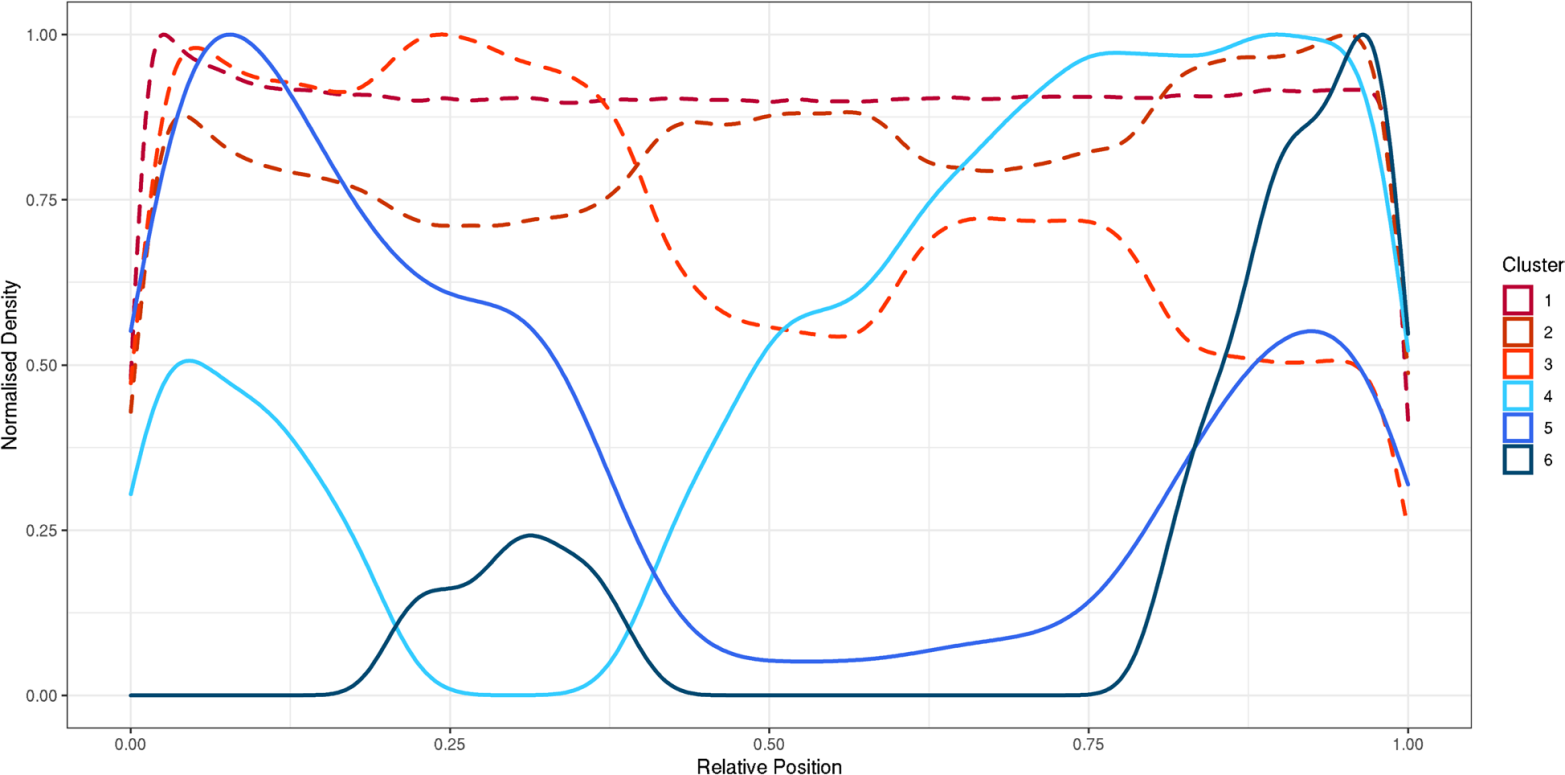
Profile of variant locations in each identified cluster. Frequency density plots of the relative position of all variants in all genes falling into each cluster. Shown are the six clusters which identified genes where variants of a particular class are present. The 7th cluster identified genes where there were no variants of a given class

We calculated the relative location of every coding variant detected in ES data from 454,773 individuals in UKB in the coding sequence (CDS) of each gene, and the relative proportion of variants grouped by consequence class in each transcript [20] (synonymous, missense, pLoF) within each quintile of the CDS. We then used Gaussian mixtures to cluster the profile of the variants of each class within the transcripts into seven clusters (Fig. 1). Of these, three clusters represented variants being distributed more-or-less uniformly throughout the CDS (clusters 1–3; Fig. 2), and one identified genes with no variants of a particular class (not shown). The remaining three clusters showed distinct patterns in the location of variants, with at least one quintile of the CDS containing zero (or very few) variants of a given variant class, and most variants either being towards the first or second half of the gene (clusters 4–6; Fig. 2, Additional file 1: Figs. S1-S2).

**Fig. 3:**
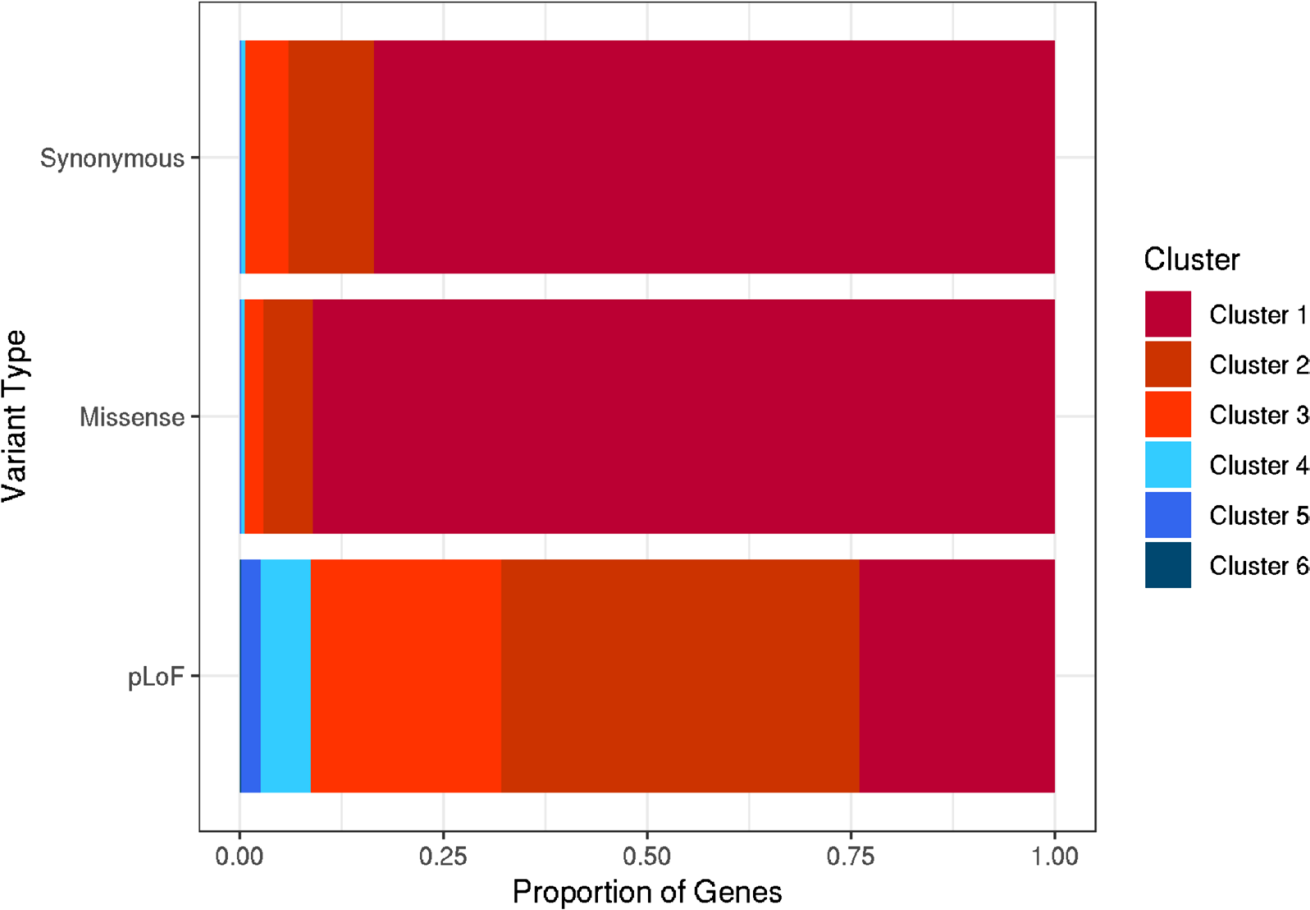
Proportion of genes falling into each cluster, separated by variant class. For each class of variant (synonymous, missense, pLoF), we present the relative proportion of genes where variants of that class were included in each of the six clusters where variants are present. The seventh cluster identifying genes with no variants of a particular class was excluded from the relative proportion calculations

**Fig. 4:**
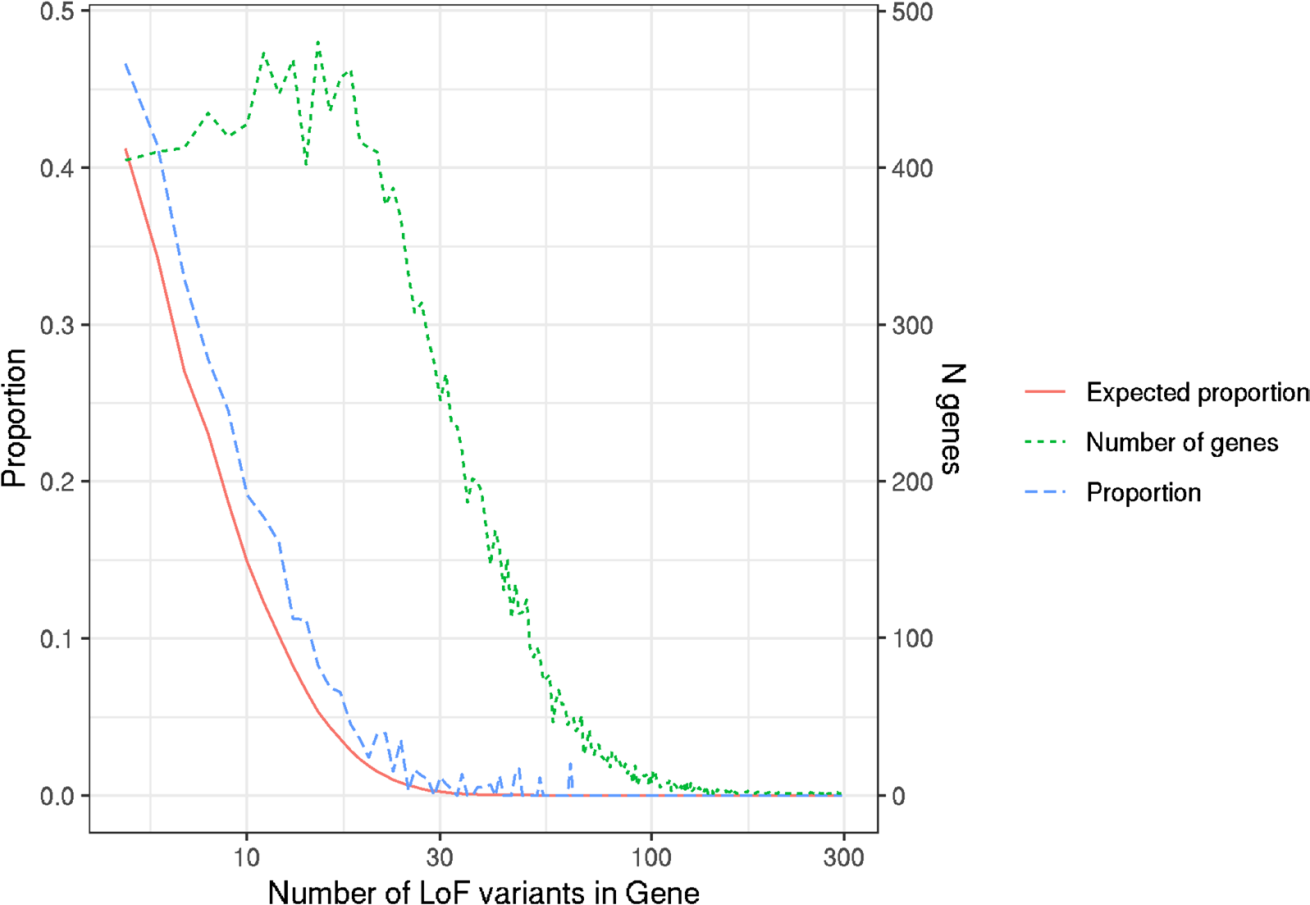
Proportions of genes in clusters 4–6 in UKB compared with simulations. The proportion of genes where pLoFs in the UK Biobank are clustered into clusters 4–6 against the number of pLoFs in that gene is shown in blue. The proportion of simulated genes where pLoFs clustered into clusters 4–6 against the number of variants in the gene is shown in red. The green line shows the number of genes with each number of pLoFs in UK Biobank. The *X*-axis is log10-transformed

We limited our analyses to transcripts with at least 5 variants of each consequence class and only considered MANE Select transcripts for each gene in our primary analysis (16,473 genes). We found that, for most genes, synonymous and missense variants fell within the uniform clusters 1–3 (99.3% and 99.4%, respectively; Fig. 3). In contrast, we found considerably more genes with pLoFs in the non-uniform clusters 4–6 (*n* = 1460, 8.9%) compared to synonymous and missense (*P* < 2.2e−16). These distinct profiles for pLoF location were not driven by the possible locations of pLoF variants, based on the underlying sequence, where only 63 genes had non-uniform distributions. Simulations showed that, whilst constrained genes were more likely to fall into the non-uniform distribution clusters, there was an enrichment of genes within these clusters compared to the expected distribution (*P* < 0.0001; Fig. 4). We excluded 114 genes with synonymous or missense variants in non-uniform clusters 4–6 from further analysis as these could be indicative of poor coverage over large regions of the gene.

### pLoF variants are more likely to be non-uniformly distributed in genes linked with autosomal dominant conditions

**Fig. 5:**
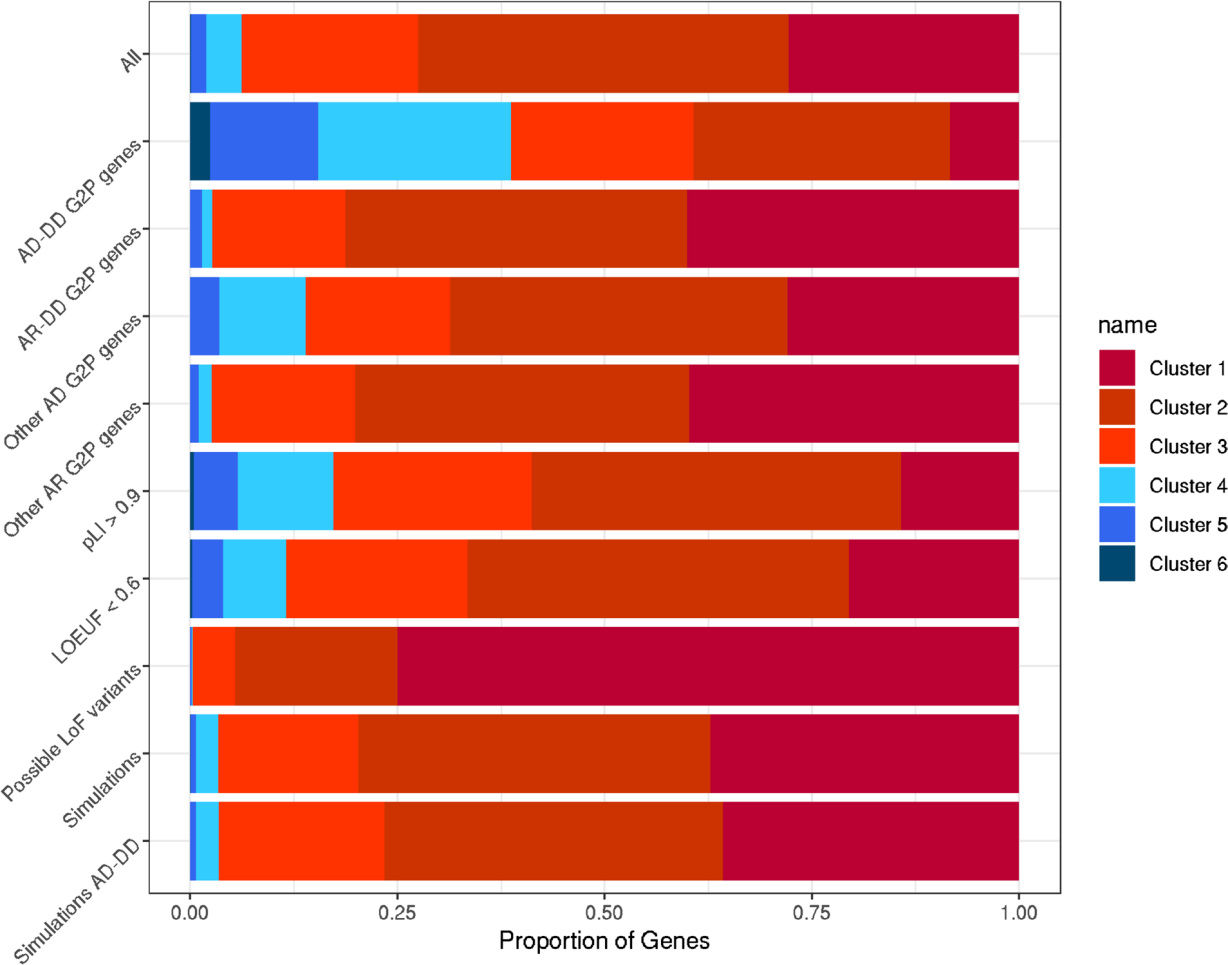
Proportion of genes with pLoFs in each cluster, including subsets of disease genes, locations of possible pLoFs, and simulation analyses. The relative proportion of genes where pLoFs are included in each of the six clusters with at least five pLoF variants is shown for different sets of genes, locations of possible pLoFs, and simulations: all (all genes with at least five pLoFs, *N* = 15,874), AD-DD G2P genes (genes where pLoFs cause developmental delay through haploinsufficiency, *N* = 217), AR-DD G2P genes (genes where pLoFs cause developmental delay through recessive mechanisms, *N* = 889), other AD G2P genes (genes where pLoFs cause adult onset diseases, including cancer syndromes and heritable cardiac, eye or skin conditions through haploinsufficiency, *N* = 98), other AR G2P genes (genes where pLoFs cause adult onset diseases through recessive mechanisms, *N* = 222), genes with high probability of LoF intolerance (pLI from gnomAD v2.1.1 [16]) scores > 0.9 (*N* = 2097), loss-of-function observed/expected upper bound fraction (LOEUF from gnomAD v2.1.1 [15]) score < 0.6 (*N* = 4475), possible LoF variants based on the underlying sequence of each gene (*N* = 15,874), simulations of all genes (simulated genes matched to the number of pLoFs in each gene in UKB, *N* = 15,874), and simulations of AD-DD genes (simulated genes matched to the number of pLoFs in AD-DD genes in UKB, *N* = 217). AD, autosomal dominant; AR, autosomal recessive

We observed that pLoF variants in UKB were more likely to be non-uniformly distributed in autosomal dominant DD genes (AD-DD) [21] versus other genes, possibly indicating regions where pLoFs are tolerated and do not cause severe disease. Of AD-DD genes, where pLoFs cause DD through haploinsufficiency (421 genes) [21], we observed 29 with no coding pLoFs in UKB and 153 with < 5 pLoFs (Additional file 2: Table S1). Of the remaining 239 AD-DD genes, pLoFs in 41.4% were non-uniformly distributed (Fig. 5), which contrasted with 3.3% for autosomal recessive DD (AR-DD) genes. Genes linked with a range of adult-onset autosomal dominant diseases (including cardiac conditions, heritable cancer syndromes, and eye disorders), were also more likely to have non-uniformly distributed pLoFs (25.3%) than other genes. Simulations showed that whilst these genes generally had fewer pLoFs than other genes, this did not explain the non-uniform distributions of pLoFs (*P* < 0.0001).

**Fig. 6:**
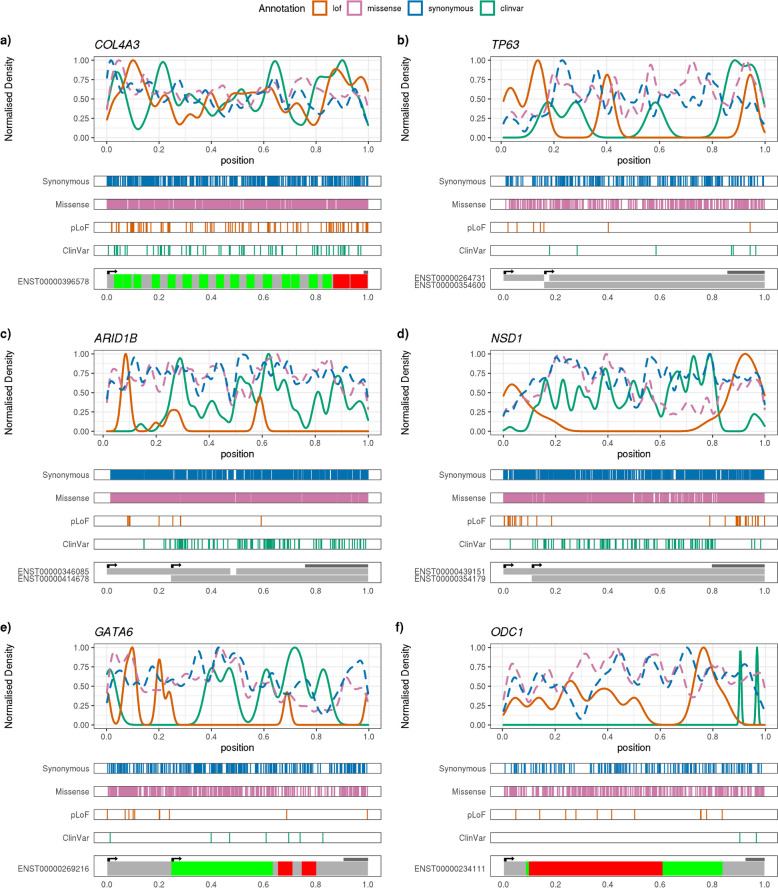
Profiles of variants of each class within selected exemplar DDG2P genes. Locations of variants of each class in UK Biobank individuals, and ClinVar pathogenic/likely pathogenic variants in *COL4A3*, *TP63*, *ARID1B*, *NSD1*, *GATA6*, and *ODC1* are shown. The top panel of each figure shows a frequency density plot of the relative position of variants of each class in UKB, plus ClinVar pathogenic/likely pathogenic variants. The middle panels show rug plots of the relative positions of each variant of each class in separate panels. The bottom panels show the locations of likely start codons (i.e. in-frame ATG), a diagram of either the relative positions of domains within the protein (for *COL4A3*, *GATA6*, *ODC1*), or a depiction of the exons included in the labelled transcript (*TP63*, *ARID1B*, *NSD1*). The location of the final exon is indicated by the dark bar above the transcript diagram

Applying the same clustering procedure to disease-causing (pathogenic/likely pathogenic) pLoF variants in ClinVar [22], we found that among 1438 genes with at least 5 such variants, only 51 (3.5%) were non-uniformly distributed. Across 156 AD-DD genes with at least 5 pLoFs in both UKB and ClinVar datasets, we found 83 (53.2%) genes where pLoFs fell into 1 of the uniform clusters in UKB, compared with 150 (96.2%) in ClinVar (2-sided binomial *P* < 2.2 × 10^−16^). The majority of genes clustered similarly in both datasets; for example, pLoF variants in *COL4A3* (associated with Alport syndrome, MIM #104200) are uniformly distributed throughout the gene in both UKB and ClinVar (Fig. 6a). In such cases, where pLoFs are uniformly distributed throughout a gene in both population and clinical datasets, this approach is not able to determine why some pLoF variants are likely to be benign whilst others are pathogenic.

### Non-uniform distributions of pLoF variants may explain incomplete penetrance through a variety of molecular mechanisms including alternative splicing and translation re-initiation

In 43.6% of genes with at least five pLoF variants in both datasets, the distribution of pLoF variants differed substantively between UKB and ClinVar (i.e., one was uniform whilst the other was non-uniform). We hypothesised that these might represent examples where variant locations could explain incomplete penetrance. We examined this list of genes for examples where the difference in distributions was robust, based on visual inspection of the underlying pLoF variant distributions and sequence data, and investigated potential mechanistic explanations.

One mechanism which might explain incomplete penetrance is the existence of alternative transcripts, where benign pLoF variants are clustered in exons that are excluded from other functional transcripts. For example, *TP63* (associated with numerous conditions, for example AEC syndrome, MIM #106260) has seven pLoFs in the MANE Select transcript in UKB, of which 5 are in early exons not present in the MANE Plus Clinical transcript (Fig. 6b). This transcript (ENST00000354600) has an alternative later start codon but contains all the ClinVar pathogenic pLoF variants. In addition to MANE Plus Clinical transcripts, which may be the most obvious candidates for alternative transcripts to explain the presence of pLoFs in apparently healthy individuals, other transcripts may show higher expression levels and explain non-penetrance of some disease genes. Other examples highlighted by our analysis include 2 large genes, *ARID1B* (associated with Coffin-Siris syndrome, MIM #135900) and *NSD1* (associated with Sotos syndrome, MIM #117550), which have 13 and 31 pLoFs in UKB, respectively. For *ARID1B*, 10/13 pLoFs fall before Met584 of the MANE Select transcript (ENST00000346085), which also corresponds to the start codon of an alternative transcript (ENST00000414678) that shows higher expression in GTeX v7 than the MANE Select transcript [23] (Fig. 6c). For *NSD1*, all the pLoFs in UKB occur either in the large last exon or the first exon of the MANE Select transcript (ENST00000439151) (Fig. 6d). The first exon is excluded from an alternative transcript, ENST00000354179, which has much higher expression levels in GTeX v7 than the MANE Select transcript. Whilst the final exon is included in both transcripts, it lies downstream of the functional domains of the protein, and since pLoFs in the final exon usually escape NMD, a functional C-terminally truncated protein could be produced. In both cases, the pLoF variants in ClinVar are fairly uniformly distributed throughout the rest of the gene, but lie primarily outside of these exons.

It is also important to consider not only alternative transcription but also translation re-initiation in explaining incomplete penetrance, as alternative start sites on the same transcript could rescue some pLoF variants (though these may not be annotated as such). For example, *GATA6* (associated with pancreatic agenesis and congenital heart defects, MIM #600001) has 10 pLoFs in UKB, of which eight are located before the first inframe ATG downstream of the canonical start codon, at Met147 (Fig. 6e). This contrasts with ClinVar variants, which all lie after Met147. Although there is only a single known transcript for this gene, it has previously been shown that *GATA6* can be produced through translation re-initiation from a downstream ATG at Met147, creating a second recognised protein isoform [24] that is shorter but still retains the functional domains. Unlike the many other AD-DD genes, the phenotypes linked with *GATA6* haploinsufficiency are both specific and severe enough that we considered it implausible they would not be recorded in the linked electronic health records of UKB participants; importantly, we note that none of the 30 carriers have any indication of either pancreatic agenesis or cardiac malformations.

### Pathogenic pLoF variants at the end of genes may point towards a gain-of-function disease mechanism

Finally, we also found a small number of AD-DD genes where pLoF variants were uniformly distributed in UKB but non-uniformly distributed in ClinVar. For example, in *ODC1* (associated with Bachmann-Bupp syndrome, MIM #165640), all 11 pLoFs in UKB occur before the penultimate exon, whilst ClinVar pathogenic variants all occur in the last or penultimate exons (Fig. 6f). Here, despite being annotated as pLoF, there is no evidence that haploinsufficiency causes disease, and pathogenic variants at the end of the gene are likely to result in a gain-of-function (GoF), for example, by causing resistance to normal protein degradation [25]. GoF mechanisms have been shown to be an important mechanism for variant pathogenicity [14].

## Discussion

Using cluster analysis, we have identified 1460 genes which show distinct patterns of pLoF location within UKB, of which 16.4% are in genes where haploinsufficiency causes monogenic diseases that are generally assumed to be highly penetrant. We have also highlighted specific examples of well-clinically characterised genes, including *GATA6* and *ARID1B*, where we were able to suggest potential molecular mechanisms that may explain the presence of pLoF variants in apparently healthy individuals. These examples show the importance of examining alternative transcription and alternative translation to understand the clinical impact of pLoFs.

Haploinsufficient genes can be divided into three groups based on the distribution of population genetic variation in UKB: (1) those where we observe too few pLoF variants to be able to cluster them effectively (37.4%), (2) those where we observe distinct non-uniform patterns of pLoF variant distribution (20.9%) and (3) those where we observe a broadly uniform distribution of pLoF variants (41.6%). For the first of these groups, the low numbers of pLoFs in UKB may be the result of haploinsufficiency in these genes being genuinely highly penetrant; for example, *STXBP1*, *MED13L*, and *PURA* (in which haploinsufficiency is associated with severe intellectual disability) [26–28] all have > 100 pLoFs in ClinVar but no coding pLoFs in UKB. For the second group, we have demonstrated how a non-uniform distribution can explain incomplete penetrance of pLoF variants in many of these genes, such as *GATA6*, *ARID1B*, and *NSD1*. The final group of genes (where we observe uniform distributions of pLoFs in UKB) is perhaps most puzzling; although a subset may exhibit patterns of pLoF variant locations that are below the resolution captured by the quintiles used in our clustering approach, this is unlikely to be the case for all of them. Similarly, although a subset may cause unrecognised or mild developmental disorders in some individuals in UKB, this is unlikely to be true for the majority given the known ascertainment bias towards healthy individuals [29]. However, some genes (such as *ODC1* and *SRCAP* [30]) contain pLoFs that cause clinically distinct DDs via different mechanisms based on their location, such as toxic GoF, with phenotypes ranging from mild to severe. The location of variants in the final exon causing disease through GoF mechanisms has previously been examined [14]; our exon-agnostic approach demonstrates an alternative method to identify such variants, and methods for predicting GoF variants do not currently exist to allow these to be systematically examined here. For other genes with uniformly distributed pLoFs, the presence of incompletely penetrant pLoF variants may instead indicate the presence of modifiers, potentially in other genes or nearby non-coding regions. Understanding the mechanisms that modify the penetrance of these genes will require sequence data on large numbers of affected individuals to compare to healthy controls and is beyond the scope of this study but would enable assessment of genotype-phenotype correlations and disease mechanisms at a sub-genic level.

Whilst our study has identified genes where distinct patterns of pLoFs point towards mechanisms that may explain incomplete penetrance, there are some notable limitations. The use of exon-agnostic quintiles to normalise the position of variants within genes means there will be patterns which are missed by our clustering approach, as their distribution is below the resolution captured by quintiles. Also, as demonstrated by the simulation analyses, there are a number of genes which will fall into clusters with distinct patterns of pLoF distribution by chance, rather than being driven by underlying biological mechanisms. Identifying these genes and separating them from those where the patterns of pLoFs are informative requires additional data and may not always be possible with high confidence. The lack of an obvious separation of the clusters in PCA space (Additional file 1: Fig. S1) despite the biological relevance of the clusters we have demonstrated here also suggests that a subset of the genes, especially at the boundaries of clusters, which may fall into clusters due to technical rather than biological reasons. However, we believe that we have demonstrated the utility of our approach, which will improve with larger datasets. Additionally, whilst none of the individuals in UKB carrying pLoFs in the genes highlighted have been diagnosed with any of the conditions in question, there may be relevant phenotypes not captured in the UKB data. Increasing the sample size would also allow us to increase the robustness of the clustering, especially for highly constrained genes with few carriers in UKB. Finally, we did not explore other genetic mechanisms or potential modifiers, such as digenic inheritance or polygenic risk, as the method outlined here is most useful for identifying candidate regions of genes associated with haploinsufficiency where pLoF variants may be benign. Other existing regional intolerance scores based on missense variants may be more appropriate for considering other mechanisms such as GoF or effects of variants on protein domains and structure.

## Conclusions

We have shown how genes associated with assumed fully penetrant childhood-onset conditions through haploinsuffiency can have regions where predicted pathogenic variants are tolerated and do not cause disease. Excluding such variants from both diagnostic pipelines and studies of disease penetrance is crucial. For example, within *GATA6* and *ARID1B*, we suggest that pLoFs occurring in the first quarter of the CDS of the MANE Select transcript (corresponding to the first 146 and 583 amino acids of the proteins, respectively) do not cause disease and should not be routinely reported diagnostically. We have also demonstrated the benefits of using regional rather than gene-wide constraint metrics to understand the potential impact of pLoF variants, which are complementary to the existing regional missense constraint metrics. Our results may be helpful in determining whether individual pLoF variants in genes associated with monogenic conditions cause disease.

## Supplementary Information

**Additional file 1.** Figs. S1 and S2 showing the clusters assigned to genes in PCA space and compared to the proportion of variants per quintile

**Additional file 2.** Table S1. Contains Table S1 showing the clusters assigned to genes, along with descriptive data on the genes

## Data Availability

Analysis code is available on GitHub doi:10.5281/zenodo.10848441

## References

[1] Beaumont RN, Wright CF. Estimating diagnostic noise in panel-based genomic analysis - ScienceDirect. Genet Med. 2022;24:2042–50. 35920826 10.1016/j.gim.2022.06.008

[2] Van Hout CV, et al. Exome sequencing and characterization of 49,960 individuals in the UK Biobank. Nature. 2020;586:749–56. 33087929 10.1038/s41586-020-2853-0 PMC7759458

[3] Klemenzdottir EO, et al. A population-based survey of FBN1 variants in Iceland reveals underdiagnosis of Marfan syndrome. Eur J Hum Genet. 2024;32:44–51. 10.1038/s41431-023-01455-0 PMC10772070 37684520

[4] Kingdom R, et al. Rare genetic variants in genes and loci linked to dominant monogenic developmental disorders cause milder related phenotypes in the general population. Am J Hum Genet. 2022;109:1308–16. 35700724 10.1016/j.ajhg.2022.05.011 PMC9300873

[5] Gardner EJ, et al. Reduced reproductive success is associated with selective constraint on human genes. Nature. 2022;603:858–63. 35322230 10.1038/s41586-022-04549-9

[6] Pizzo L, et al. Rare variants in the genetic background modulate cognitive and developmental phenotypes in individuals carrying disease-associated variants. Genet Med Off J Am Coll Med Genet. 2019;21:816–25. 10.1038/s41436-018-0266-3 PMC6405313 30190612

[7] Kurki MI, et al. Contribution of rare and common variants to intellectual disability in a sub-isolate of Northern Finland. Nat Commun. 2019;10:410. 30679432 10.1038/s41467-018-08262-y PMC6345990

[8] Kingdom R, et al. Genetic modifiers of rare variants in monogenic developmental disorder loci. medRxiv. 2022. https://www.medrxiv.org/content/10.1101/2022.12.15.22283523v1. 10.1038/s41588-024-01710-0 PMC11096126 38637616

[9] MacArthur DG, et al. A systematic survey of loss-of-function variants in human protein-coding genes. Science. 2012;335:823–8. 10.1126/science.1215040 PMC3299548 22344438

[10] de Klerk, E. & ‘t Hoen, P. A. C. Alternative mRNA transcription, processing, and translation: insights from RNA sequencing. Trends Genet. 31, 128–139 (2015). 10.1016/j.tig.2015.01.001 25648499

[11] Dietz HC, Kendzior RJ. Maintenance of an open reading frame as an additional level of scrutiny during splice site selection. Nat Genet. 1994;8:183–8. 7842017 10.1038/ng1094-183

[12] Dyle MC, Kolakada D, Cortazar MA, Jagannathan S. How to get away with nonsense: mechanisms and consequences of escape from nonsense-mediated RNA decay. WIREs RNA. 2020;11:e1560. 31359616 10.1002/wrna.1560 PMC10685860

[13] Wethmar K. The regulatory potential of upstream open reading frames in eukaryotic gene expression. WIREs RNA. 2014;5:765–8. 10.1002/wrna.1245 24995549

[14] Coban-Akdemir Z, et al. Identifying genes whose mutant transcripts cause dominant disease traits by potential gain-of-function alleles. Am J Hum Genet. 2018;103:171–87. 30032986 10.1016/j.ajhg.2018.06.009 PMC6081281

[15] Karczewski KJ, et al. The mutational constraint spectrum quantified from variation in 141,456 humans. Nature. 2020;581:434–43. 32461654 10.1038/s41586-020-2308-7 PMC7334197

[16] Lek M, et al. Analysis of protein-coding genetic variation in 60,706 humans. Nature. 2016;536:285–91. 10.1038/nature19057 PMC5018207 27535533

[17] Samocha, K. E. et al. Regional missense constraint improves variant deleteriousness prediction. 148353 Preprint at https://doi.org/10.1101/148353 (2017).

[18] Havrilla JM, Pedersen BS, Layer RM, Quinlan AR. A map of constrained coding regions in the human genome. Nat Genet. 2019;51:88–95. 30531870 10.1038/s41588-018-0294-6 PMC6589356

[19] Eggertsson HP, et al. GraphTyper2 enables population-scale genotyping of structural variation using pangenome graphs. Nat Commun. 2019;10:5402. 31776332 10.1038/s41467-019-13341-9 PMC6881350

[20] McLaren W, et al. The Ensembl variant effect predictor. Genome Biol. 2016;17:122. 27268795 10.1186/s13059-016-0974-4 PMC4893825

[21] Thormann A, et al. Flexible and scalable diagnostic filtering of genomic variants using G2P with Ensembl VEP. Nat Commun. 2019;10:2373. 31147538 10.1038/s41467-019-10016-3 PMC6542828

[22] Landrum MJ, et al. ClinVar: improving access to variant interpretations and supporting evidence. Nucleic Acids Res. 2018;46(D1):1062–7. 10.1093/nar/gkx1153 PMC5753237 29165669

[23] The GTEx Consortium atlas of genetic regulatory effects across human tissues | Science. https://www.science.org/doi/10.1126/science.aaz1776. 10.1126/science.aaz1776 PMC7737656 32913098

[24] Chia CY, et al. GATA6 cooperates with EOMES/SMAD2/3 to deploy the gene regulatory network governing human definitive endoderm and pancreas formation. Stem Cell Rep. 2019;12:57–70. 10.1016/j.stemcr.2018.12.003 PMC6335596 30629940

[25] Schultz CR, et al. Biochemical features of primary cells from a pediatric patient with a gain-of-function ODC1 genetic mutation. Biochem J. 2019;476(14):2047–57. 10.1042/BCJ20190294 31249027

[26] Hunt D, et al. Whole exome sequencing in family trios reveals de novo mutations in PURA as a cause of severe neurodevelopmental delay and learning disability. J Med Genet. 2014;51:806–13. 25342064 10.1136/jmedgenet-2014-102798 PMC4251168

[27] Protein structure and phenotypic analysis of pathogenic and population missense variants in STXBP1 - Suri - 2017 - Molecular Genetics & Genomic Medicine - Wiley Online Library. Mol Genet Genomic Med. 2017;5:495–507. 10.1002/mgg3.304 PMC5606886 28944233

[28] Adegbola A, et al. Redefining the MED13L syndrome. Eur J Hum Genet. 2015;23:1308–17. 25758992 10.1038/ejhg.2015.26 PMC4592099

[29] Schoeler T, et al. Participation bias in the UK Biobank distorts genetic associations and downstream analyses. Nat Hum Behav. 2023;7:1216–27. 37106081 10.1038/s41562-023-01579-9 PMC10365993

[30] Rots D, et al. Truncating SRCAP variants outside the Floating-Harbor syndrome locus cause a distinct neurodevelopmental disorder with a specific DNA methylation signature. Am J Hum Genet. 2021;108:1053–68. 33909990 10.1016/j.ajhg.2021.04.008 PMC8206150

