# Supplementary material for: Clustering of predicted loss-of-function variants in genes linked with monogenic disease can explain incomplete penetrance
Source: Genome Med. 2024 Apr 26;16:64. doi: 10.1186/s13073-024-01333-4 (PMC11046769; doi:10.1186/s13073-024-01333-4)

**Figure S1:** Principal components scatter plots, with genes coloured based on the clusters they were grouped into.


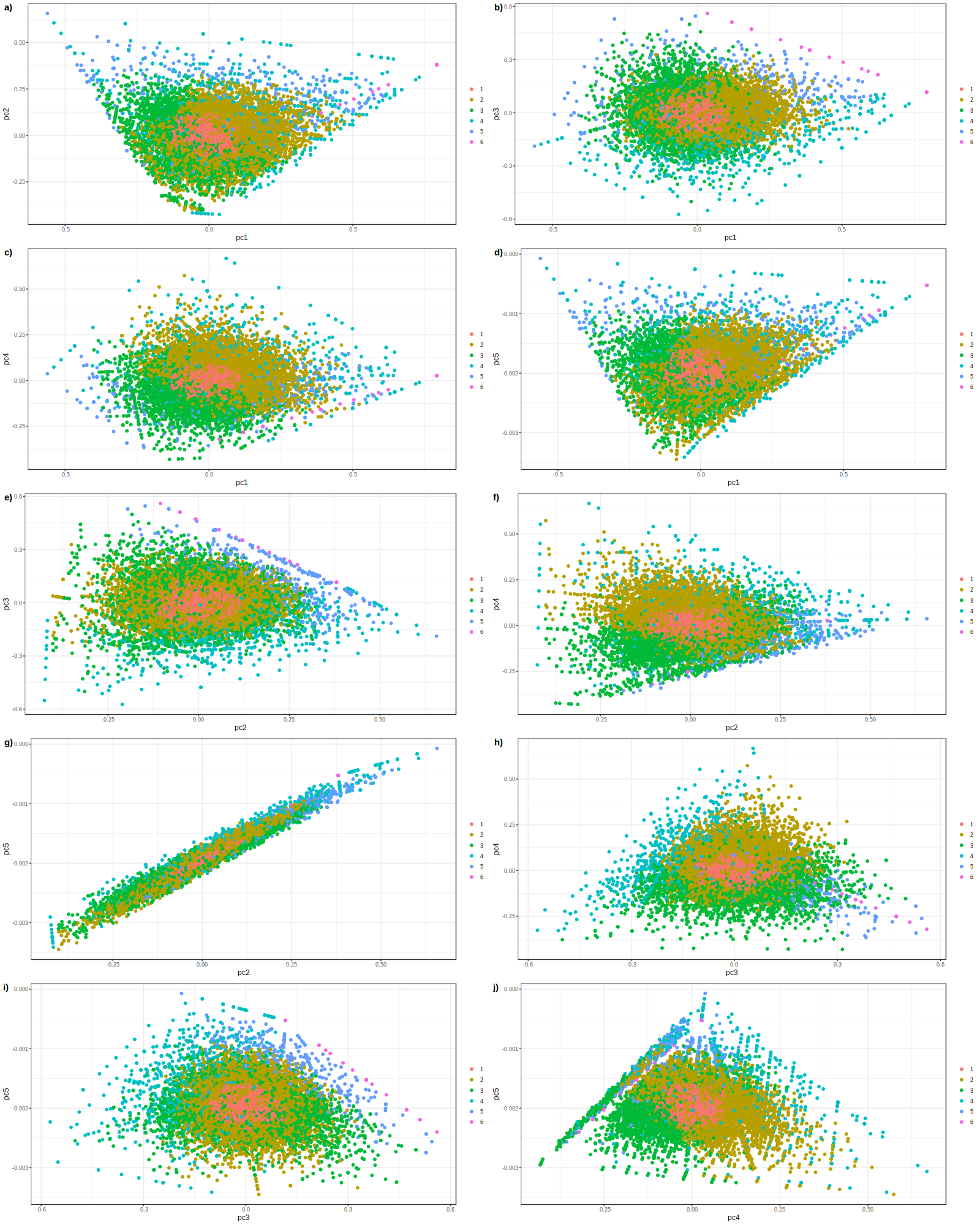


**Figure S2:** Scatter plots showing the proportion of variants within each quintile in each MANE Select transcript, with genes coloured based on the clusters they were grouped into.


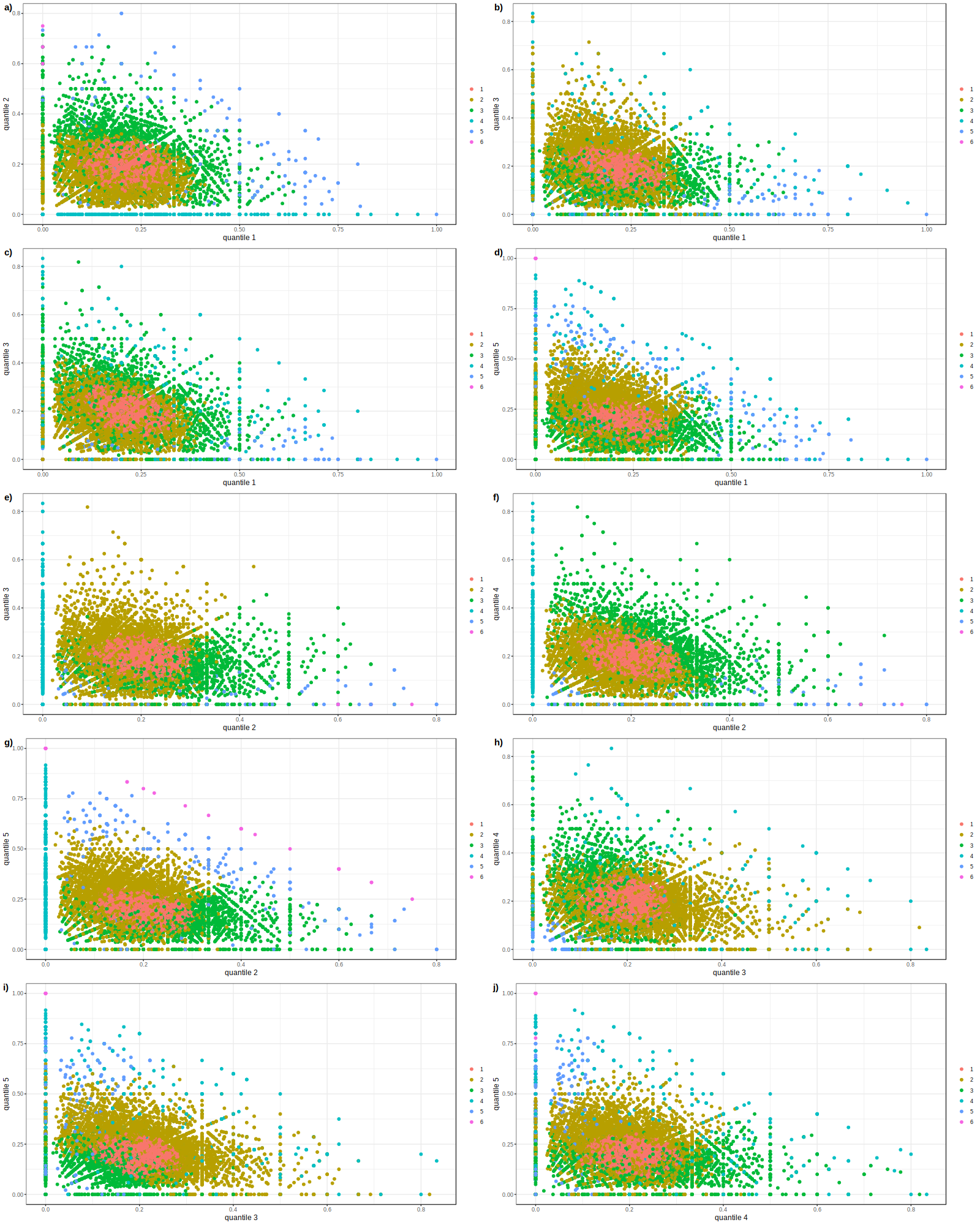

Supplement: Supplementary file 1 — Additional file 1. Figs. S1 and S2 showing the clusters assigned to genes in PCA space and compared to the proportion of variants per quintile [file 13073_2024_1333_MOESM1_ESM.docx]
